# Sexual Violence against Women in Germany: Prevalence and Risk Markers

**DOI:** 10.3390/ijerph15081613

**Published:** 2018-07-30

**Authors:** Deborah F. Hellmann, Max W. Kinninger, Sören Kliem

**Affiliations:** 1 University of Applied Administrative Sciences NRW, 47269 Duisburg, Germany; 2 Department of International Public Law and Comparative Law, Albert-Ludwigs-University Freiburg, 79085 Freiburg im Breisgau, Germany; max.kinninger@gmail.com; 3 Criminological Research Institute of Lower Saxony, 30161 Hannover, Germany; soeren.kliem@kfn.de

**Keywords:** rape, rape victim, rape survivor, victimization risk, child maltreatment, conditional inference trees, representative victim survey

## Abstract

Previous research has repeatedly shown that gender-based violence affects a considerable proportion of women in any given population. Apart from providing current estimates of the prevalence of sexual violence against women in Germany, we identified specific risk markers applying an advanced statistical method. We analyzed data from a survey of *N* = 4450 women representative of the German population, conducted by the Criminological Research Institute of Lower Saxony in 2011. Lifetime prevalence for experiencing sexual violence was 5.4% for women aged 21–40 years (five-year prevalence: 2.5%). Non-parametric conditional inference tree (C-Tree) analyses revealed that physical and sexual abuse during childhood as well as being divorced, separated, or widowed was the most informative constellation of risk markers, increasing the five-year prevalence rate of experienced sexual violence victimizations up to 17.0%. Furthermore, knowing about the official penalization of marital rape was related to a lower victimization risk for women without a history of parental violence. Possible explanations for these findings as well as implications for future research are critically discussed.

## 1. Introduction

Experiencing gender-based violence can have tremendous consequences for the survivor’s mental and physical functioning, increasing the risks of poor health in general and of certain conditions, such as depression, Post-Traumatic Stress Disorder, anxiety, and chronic pain in particular [1,2,3]. Research has consistently demonstrated that sexual violence victimizations are in fact not an exception, but do affect a considerable proportion of women in any given population [4,5]. According to the results of the National Intimate Partner and Sexual Violence Survey 2010, 9.4% (i.e., approximately 11.2 million) of women in the U.S. have at least once in their lifetime been raped by an intimate partner [6]. Another 15.9% of the surveyed women (i.e., approximately 19 million) reported that they had been sexually victimized in some other form (e.g., “unwanted sexual contact”; [6], p. 13, also see [7,8]).

In a comprehensive study by the Fundamental Rights Agency (FRA) assessing violence against women aged 18 to 74 years in 28 EU Member States, 5% of more than *N* = 42,000 surveyed women indicated that they had been raped at least once since the age of 15 years [5]. Overall, 11% of the respondents indicated that they had experienced some form of sexual violence since the age of 15 years. Approximately 2% of the surveyed women had experienced sexual violence in the last 12 months—these data lead to estimates of 3.7 million women in the EU with a history of experienced sexual violence (also see [9]). However, prevalence rates of sexual violence obviously vary considerably—likely due to various reasons such as methodological differences in assessing experienced sexual violence as well as differences in samples and in (legal) definitions, which can be diverging within and between countries and may change over time [5,10,11].

Because only a minority of incidences of sexual violence are reported to the police and result in a conviction [5,12,13,14], large-scale epidemiological studies (e.g., [4,5,6]) are of major importance in this regard. Such studies survey non-victims, victims who disclosed to the police, as well as victims who did not turn to the legal authorities and they mostly cover a broad range of victimization accounts. However, such reports often remain on the surface of descriptively informing about prevalence rates and certain victimization realities. Whereas these accounts are of major importance in order to raise public awareness and are necessary to understand the burden of the problem in different populations [15], it seems equally important to dig deeper into the matter—for example by applying statistically advanced analyses of specific factors that might increase the risk of experiencing gender-based violence (e.g., [16,17,18,19]).

Krahé and colleagues [20], for instance, recently collected and analyzed data on youth sexual aggression in ten European countries, using identical materials and applying the same definition of sexual violence in all countries. These authors found in their convenience samples varying prevalence rates of sexual violence against young women between 20% (Lithuania) and 52% (the Netherlands). This study illustrates the usefulness of consistent conceptualizations of sexual violence victimization in research that aims at comparing the distribution of sexual violence victimizations between countries and over time. Furthermore, Krahé and colleagues [20] applied a multilevel approach combining individual-level and country-level predictors of sexual aggression in order to further analyze the varying prevalence rates. However, while research increasingly aims at applying statistically advanced methods to the distribution and risk factors associated with sexual violence victimizations (e.g., [16,21]), the aforementioned study [20] is one of the few examples including European countries, such as Germany, for example. Beyond that, investigating prevalence and risk markers of sexual violence against women in Germany is especially interesting as to the best of our knowledge (potential) victims’ knowledge about the current legal situation regarding marital rape has not yet been taken into account.

Thus, the purpose of the present study is to obtain current estimates of the prevalence of sexual violence against women in Germany. Going beyond previous research by applying non-parametric conditional inference tree (C-Tree) analyses [18,19], we specifically take into account factors that have been associated with an increased victimization risk in previous research (i.e., experienced child sexual abuse, experienced parental violence in childhood, and sociodemographic variables, such as age, educational degree, household size, origin, relationship status, and residential area) as well as potential protective factors, such as knowing about the penalization of marital rape, that have not been accounted for in the literature.

### 1.1. Risk Markers for Sexual Violence Victimizations

Basically, sexual violence victimizations can happen to everybody [2,4,5,22]—although women are definitely at a higher risk of experiencing sexual violence in their lives as compared to men [6,23]. However, previous research has identified certain individual and contextual factors associated with an increased risk of becoming a survivor of sexual violence. Some respective results that pose the basis for our own reasoning are presented in the following sections. In this regard, it is important to note that obviously no blame is to be ascribed to the survivors themselves. Research on rape myths acceptance [24], for example, has repeatedly shown, that victims of sexual violence are often held responsible for the victimization (i.e., victim blaming)—especially in cases of substance use in association with dating [25]. Importantly, women’s behaviors are not causally related to experienced sexual violence, as offenders are responsible for victimizations and the victims are not.

#### 1.1.1. Child Sexual Abuse and Parental Violence

Experienced child sexual abuse (CSA) constitutes one of the most consistently identified risk markers for sexual revictimization later in life [26,27]. Trickett and colleagues [28], for example, reported that “sexually abused females were almost twice as likely to have experienced sexual revictimization […] compared to victimization rates reported by comparison females” (p. 463). Similarly, according to a review by Classen and colleagues [29], experienced CSA more than doubled females’ risks for later sexual assault.

Regarding the nature of this link, previous research proposed several potential explanations and mediators, such as the number of sexual partners [30], risky sexual behavior [31], or substance abuse [29,32]. In this regard, Noll and colleagues [33] argued that experiencing sexual violence as a child shapes concepts of and attitudes towards sexuality in a dysfunctional way. Accordingly, it has been found that child sexual trauma is paralleled by more sexualized attitudes and behaviors [34,35,36], which, in turn, could contribute to an increased risk of sexual revictimization. Hillis and colleagues [34], for example, found that the proportion of women with a history of sexual abuse who had 30 or more sexual partners was more than twice as high compared to women without a history of sexual abuse.

Apart from experienced CSA, a growing body of research indicates that other forms of traumatic childhood experiences, such as physical and emotional abuse or neglect [27,29], can increase the victimization risk. Widom and colleagues [37], for example, showed that reports of CSA, parental violence, and neglect were associated with an increased risk of experiencing physical or sexual violence later in life (also see [38]). Similarly, Zurbriggen and colleagues [39] found a positive correlation between childhood emotional abuse and later sexual victimization. In a related vein, Symons and colleagues [40] reported that women who had been sexually victimized since the age of 16 years indicated significantly more observed violence between their parents as well as experienced violence by at least one parent against themselves.

In sum, these exemplary findings together with the aforementioned results on the effects of CSA imply that growing up in a (sexually) violent family environment constitutes a severely increased risk of experiencing sexual violence in adolescence and adulthood, although the mediating and/or moderating processes in this regard remain comparatively understudied. Furthermore, it has been criticized, for instance, that the overlap of childhood sexual and physical abuse needs to be considered when examining revictimization risks [41,42].

#### 1.1.2. Sociodemographic Variables

Apart from prior victimization history, previous research has taken into account sexual violence survivors’ current personal characteristics and behaviors, thereby frequently referring to lifestyle-exposure theory [43] and routine activity theory [44] in order to theoretically account for relations between personal characteristics and victimization risks of sexual violence [45,46,47].

Wittebrood and Nieuwbeerta [48], for instance, found that unmarried (as compared to married and cohabiting) persons in a representative Dutch sample had a higher victimization risk for experiencing sexual offenses. Similarly, Siddique [49] reported that independent of the victim–offender relationship (stranger, acquaintance, or intimate partner), women who had never been married as well as separated and divorced women had an increased risk of sexual victimization (as compared to married women). Furthermore, living alone was a significant risk factor for experiencing sexual violence (also see [38,45]). Relating her findings to the lifestyle-routine activities framework, the author argues that as “unmarried women are more likely to participate in daily routines unaccompanied by other household members, they may be perceived more frequently by motivated offenders to be suitable targets without adequate guardianship.” [49]. However, as with the effects of sexual and physical abuse in childhood, the effects of relationship status and household size need to be statistically disentangled in order to specify risk markers for sexual violence victimizations.

Besides marital status and household size, other sociodemographic characteristics, such as age or educational background [49,50,51,52], have been linked to experienced sexual victimizations. However, dependencies between the diverse variables have to be taken into account when relating them to victimization risks (e.g., single women are more likely to be living on their own than married women). Regarding their sociodemographic background, we take into account participants’ age, educational degree, household size, origin, relationship status, and residential area in the present study.

#### 1.1.3. Knowledge About the Penalization of Marital Rape

For many years, marital rape was not considered a criminal offense within German law. Victims of marital rape had the sole option of prosecuting their spouse due to coercion, battery, or libel. After long-term debates in politics and science and accompanied by numerous expansions of the offense, marital rape was finally made a punishable offence in 1997 [53,54,55].

In the domain of parental violence, legal changes have already been shown to influence victimization as well as perpetration risks. Hellmann and colleagues [42], for example, found that the risk of the intergenerational transmission of parental violence was significantly lower for caregivers who currently cared for at least two children and who knew about the abolition of parents’ right to use corporal punishment in Germany. Furthermore, knowledge about the abolition of parents’ right to use corporal punishment was associated with a lower prevalence of experienced severe parental violence [23]. In a related vein, Bussmann and colleagues [56] showed that knowledge regarding the abolition of parents’ rights of corporal punishment was associated with a lower risk of exerting respective behavior.

Previous research has already associated the legal change regarding marital rape with a general decline in sexual violence against women. Using identical materials and matching the respective samples, Hellmann and Pfeiffer [13] found that the five-year prevalence of sexual violence victimizations had nearly halved from 1992 to 2011. Importantly, this decline pertained to sexual violence victimizations inside and outside the victims’ family and household. That is, (attempted) marital rape has decreased, too. Although the present paper focusses on victimization instead of perpetration, we explore whether knowledge about the penalization of marital rape in Germany might, as a protective factor, be associated with a decreased victimization risk. This reasoning pertains to the rationale that making marital sexual violence a punishable offense is accompanied by a general attitudinal change regarding sexual violence in society. However, if so, the association has to be due to mediating mechanisms as legal knowledge cannot causally be related to a lower victimization risk.

The protective function of knowing about the penalization of marital rape might partly be due to a higher educational level. Furthermore, similarity within social networks and dyads may play a role in this regard. Similarity is a determinant of attraction; this may pertain to attitudes, beliefs, and other personality variables, such as intelligence, for example [57]. Thus, women who are aware of the legal situation regarding marital rape more likely encounter and socialize with men who also possess this knowledge and who, for instance, share their attitudes regarding women’s right to “say no”. As most sexual violence happens in the victims’ close social environment with most offenders being (ex-)partners, spouses, and acquaintances of the victim [8,23], knowing about the legal situation regarding marital rape may have a protective function. Additionally, we assume that women who know about the penalization of marital rape may feel more entitled to actively refuse forced intercourse by their spouses, as they are aware of the fact that marital exemption has been eliminated from German law.

### 1.2. Previous Research on Sexual Violence Against Women in Germany

Because there is no periodical national German crime victimization survey, current estimates of the epidemiology of sexual violence against women in Germany based on representative samples are not easily accessible [10]. In 2003, the German Federal Ministry for Family Affairs, Senior Citizens, Women and Youth commissioned a study with a nationally representative community sample of women aged 16 to 85 years [58]. Overall, the lifetime prevalence rate of experienced sexual violence since the age of 16 years was 13%. Furthermore, 7% of those women who had previously lived in an intimate relationship or were currently living in such a relationship gave accounts of experienced sexual intimate partner violence. A previous study, conducted by the Criminological Research Institute of Lower Saxony in 1992, revealed a lifetime prevalence rate for sexual violence victimizations of 8.6% in total, referring to a representative sample of women aged 16 to 85 years in Germany [59]. Remarkably, the vast majority of these victimizations had happened in the survivors’ close familial environment. A more recent study revealed one-year prevalence rates of sexual violence victimization of 0.6% (males) and 1.2% (females) in a sample of *N* = 2422 respondents aged 18 years and above [60].

As already stated above, it is important to note that in Germany marital rape was only legally penalized as “rape” from 1997 on. However, since then, the prevalence of experienced sexual violence has already decreased [13]. Furthermore, alongside with the political and social debate about whether sexual coercion by spouses should be implemented in §177 of the German Penal Code (i.e., sexual assault and rape), the public awareness for this deed has risen. As its condemnation has been affirmatively pronounced in public, this might help to prevent (marital) sexual victimization [61]. Thus, knowledge about the legal situation and—from the perspective of married women—one’s own rights may have a protective effect against respective victimization (of course, this link could also be negatively framed, that is, not knowing about one’s own rights posits a risk factor for sexual violence victimizations).

### 1.3. The Present Research

For the German population, empirical findings on sexual violence against women based on large-scale epidemiological studies are comparably sparse. Estimates from previous surveys suggest that about every seventh woman in Germany will experience sexual violence during her life [58]. Moreover, further research is needed regarding specific markers that are associated with victimization risks—thereby accounting for diverse predictor variables and complex interactions simultaneously [17].

In the present study, we intend to address these shortcomings and utilize data from a nationwide German survey conducted by the Criminological Research Institute of Lower Saxony in 2011 for our analyses. Apart from presenting current prevalence estimates based on a nationally representative quota sample of women aged 21 to 40 years, we aim at clarifying which persons are especially at risk of experiencing sexual violence. More specifically, we expect that prior victimization in terms of CSA and parental violence as well as current sociodemographic characteristics, namely being divorced or separated and living alone, constitute crucial risk markers for experiencing sexual violence victimizations in adolescence and adulthood. Furthermore, awareness of the current legal situation regarding marital rape (i.e., knowing that marital rape is legally penalized) is assumed to protect potential victims against sexual victimization.

## 2. Method

### 2.1. Participants

For the purpose of our analyses, we reverted to a dataset of a large-scale survey from 2011 conducted by the Criminological Research Institute of Lower Saxony [23]. The original dataset consisted of *N* = 11,428 participants aged 16 to 40 years. Since we aimed at examining *sexual violence against women in adolescence and adulthood*, we restricted the sample to a subset of *N* = 4450 women aged 21 to 40 years. In doing so, we ensured that cases of sexual violence victimizations before the age of 16 years (i.e., cases of CSA) were not taken into account in reports of the presently referred to five-year prevalence rates.

According to national census statistics, this subsample is representative of the German non-institutionalized population (i.e., persons living in a private household), with respect to the quota characteristics federal state, urban-rural distribution, age (21 to 40 years), gender, educational degree, household size, and immigrant background. Besides German women without immigrant background, the sample included women with Turkish and Russian immigrant background, as these three groups constitute the three biggest ethnicities in Germany. “Immigrant background” was defined according to the definition of the German Federal Statistical Office: If persons themselves or at least one parent had the Turkish or Russian citizenship at birth, they were defined as possessing a Turkish or Russian immigrant background, respectively. As combined quotas were also applied within the sampling procedure, the sample is not only representative of the German population (with respect to the aforementioned quota characteristics) in total, but also of the population of the specific federal states, for example.

Participants had a mean age of *M* = 30.64 (*SD* = 5.97; 21–30 years: 49.8%, 31–40 years: 50.2%). A total of 10.5% interviewees had a Russian immigrant background and 8.5% possessed a Turkish background. Importantly, both groups with immigrant backgrounds were oversampled in order to be able to compare them with the much larger group of interviewees without immigrant background. This oversampling was accounted for by statistical weighting whenever required, so that overall representativeness (according to the aforementioned quota characteristics) was established. Half of the participants were unmarried (51.8%), 38.2% were married, and 10.0% indicated that they were divorced, separated, or widowed at the time of the survey. In sum, 24.0% of the surveyed women had a lower or no educational degree (i.e., a low level of education), 37.6% indicated that they possessed a Certificate of Secondary Education or middle school degree (i.e., a medium level of education), and 38.4% held an advanced technical certificate, the German high school diploma, or a university degree (i.e., a high level of education). At the point of data collection, 35.0% of the surveyed women described their current residential area as more rural, 47.1% as more urban, and 17.8% indicated living in a metropolitan area. With 82.3%, the overall majority of the interviewees was living together with other persons at the time of data collection (26.3% were living with one other person, 24.2% with two, 22.3% with three, and 9.6% with four or more), opposed to 17.7% of the respondents living on their own.

### 2.2. Materials and Procedure

Participants were recruited by a specialized institute according to the aforementioned characteristics. Prior to working on the self-administered questionnaire, participants’ demographic information was obtained in a face-to-face interview [17].

#### 2.2.1. Sexual Violence

Lifetime prevalence of experienced sexual violence was assessed with a dichotomous item: “Has anybody ever coerced you by physical force or by threat of force to engage against your will in sexual intercourse or similar activities, or attempted to do so?” (0 = “no”, 1 = “yes”). If participants affirmed that this had happened within the previous five years (five-year prevalence), they were asked to provide further details on the victimization circumstances (see [23]).

#### 2.2.2. Parental Violence

Experienced parental violence before the age of 16 years was measured using a German version [62] of the subscales “minor violence” and “severe violence” of the Conflict Tactics Scales (CTS) [63]. Although the CTS have frequently been criticized [64,65,66], they represent an internationally widespread survey instrument that aims at assessing conflicts in close relationships [67]. The subscale “minor violence” includes three items (e.g., throwing things at the other person) and the subscale “severe violence” seven items (e.g., beating the other person up).

For the present purpose, participants indicated separately for father, mother, and other primary caregivers for each item whether they had experienced respective behavior on a scale from 1 (“never”) to 5 (“very often”). Their answers were coded into 0 = “no parental violence” (i.e., no incident of any physical violence by father, mother, or other primary caregiver before the age of 16 years), 1 = “‘minor’ parental violence” (i.e., at least one incident of “minor” physical violence by father, mother, or other primary caregiver before the age of 16 years), and 2 = “severe parental violence” (i.e., at least one incident of severe physical violence by father, mother, or other primary caregiver before the age of 16 years).

#### 2.2.3. Contact CSA

As described by Posch and Bieneck [68], participants indicated with respect to seven different scenarios involving various sexually abusive behaviors whether these had happened to them on a scale from 1 (“never”) to 6 (“several times a week”). For the purpose of the present study, their responses regarding those five scenarios were taken into account, that involved physical contact between the offender and the child. That is, for example, “How often in your childhood/adolescence (before the age of 16 years) has it happened to you that a person who was at least 5 years older than yourself has asked you to touch his/her genitals or to otherwise sexually arouse himself/herself by hand or mouth?”. Responses regarding these five scenarios were coded into 0 = “no incident of contact CSA” and 1 = “at least one incident of contact CSA”. Accordingly, contact CSA was identified if at least one of the five sexually abusive behaviors including physical contact had happened to the respondent before the age of 16 years at least once. More specifically, contact CSA in this study included having been “asked to touch the perpetrator sexually, the perpetrator touching the victim’s genitals, vaginal or anal penetration of the victim by finger, tongue, object or penis, or oral penetration with the penis” ([68], pp. 117–118).

#### 2.2.4. Knowledge about the Penalization of Marital Rape

In order to discover whether respondents knew that marital rape is a crime, they were asked to answer the item “Do you know that rape in a marriage is just as prosecutable as rape by any other person?” (0 = “no”, 1 = “yes”).

### 2.3. Missing Values and Statistical Procedures

For the treatment of missing values, we used the R package MICE (Multivariate imputation by chained equations) [69]. Missing data (missing values per variable: 0–1.8%) were treated by applying chained equation modeling [70] and including the following variables: age, educational degree, household size, origin, relationship status, and residential area. Descriptive statistics including missing values for all variables used in the C-Tree analyses are presented in Table 1.

In order to identify risk markers associated with sexual violence victimizations, we used non-parametric C-Tree analyses based on the principle of recursive partitioning [17,18,19]. As a non-parametric modelling technique, C-Tree analyses can be applied to a large set of potential predictor variables in order to compute complex interactions simultaneously. Using a permutation test framework, the C-Tree algorithm tests the global null hypothesis of independence between any predictor (e.g., sociodemographic variables or prior victimization history) and the response variable (e.g., five-year prevalence of sexual violence). If the null hypothesis is rejected, the input variable which is most strongly associated with the response variable is chosen and a binary split of this variable is implemented. Steps are recursively repeated until the hypothesis is rejected at a stop criterion based on univariate *p* < 0.001. Permutation tests derive *p*-values from sample-specific permutation distributions of the test statistics.

## 3. Results

### 3.1. Prevalence Rates and Bivariate Analyses

All prevalence rates reported in this paragraph are statistically weighted to ensure representativeness. Accounts of different sample sizes are due to this statistical weighting.

The lifetime prevalence rate for sexual violence victimizations was 5.4%, the five-year prevalence was 2.5%. The majority of the respondents (52.4%) had never experienced any form of physical parental violence, whereas 33.2% reported “minor” parental violence and 14.4% had additionally experienced severe parental violence in their childhood. The prevalence of contact CSA was 7.8%. Most of the respondents (87.8%) indicated that they knew “that rape in a marriage is just as prosecutable as rape by any other person”, whereas only 12.2% reported that they did not know about the penalization of marital rape.

Bivariate analyses revealed several correlations between the five-year prevalence of sexual violence and the proposed predictor variables: Women who lived on their own (4.0% vs. 2.2%), χ² (1, *N* = 4055) = 8.22, *p* = 0.004, *Φ* = 0.05, those who were divorced, separated, or widowed (6.5% vs. 2.1%), χ² (1, *N* = 4055) = 29.37, *p* < 0.001, *Φ* = 0.09, as well as those who had a low level of education (3.5%; medium level of education: 1.7%; high level of education: 2.6%), χ² (2, *N* = 4054) = 7.70, *p* = 0.021, *Cramér’s V* = 0.04, had a significantly higher victimization risk. Age (21–30 years: 2.8%; 31–40 years: 2.2%), origin (no immigrant background: 2.5%; Turkish background: 2.0%; Russian background: 2.3%), and residential area (rural: 2.6%; urban: 2.4%; metropolitan: 2.7%) were not related to experienced sexual violence, all *p*s > 0.178. However, both experienced contact CSA (7.5% vs. 2.1%), χ² (1, *N* = 4054) = 36.31, *p* < 0.001, *Φ* = 0.10, as well as experienced parental violence (no violence: 1.0%; “minor” violence: 3.3%; severe violence: 6.0%), χ² (2, *N* = 4054) = 51.25, *p* < 0.001, *Cramér’s V* = 0.11, correlated positively with the (re)victimization risk. Furthermore, not knowing about the penalization of marital rape (4.6% vs. 2.2%) was related to a significant higher five-year prevalence rate of sexual violence, χ² (1, *N* = 4055) = 10.72, *p* = 0.001, *Φ* = −0.05.

### 3.2. C-Tree Analyses

By applying C-Tree analyses, we further examined potential risk markers for experiencing sexual violence (i.e., five-year prevalence rates) while simultaneously taking into account their interactions. We included respondents’ age, educational degree, household size, origin, relationship status, residential area as well as experienced parental violence, contact CSA, and self-reported knowledge about the penalization of marital rape as predictors in these analyses. Effect sizes (relative risks) for each predictor are provided in Table 2.

**Figure 1:**
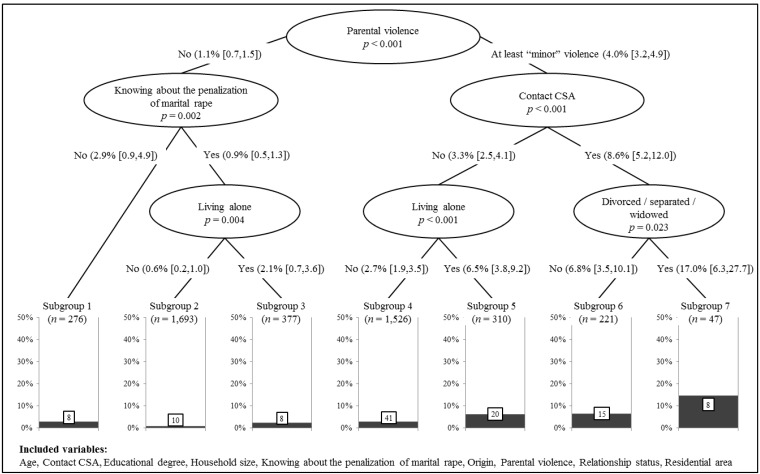
Conditional inference tree plot predicting five-year prevalence of sexual violence by age, educational degree, household size, origin, relationship status, residential area, contact CSA, parental violence, and knowledge about the penalization of marital rape (*N* = 5450). Note: Paths are labeled with the probability of experiencing sexual violence in the respective step including 95% confidence intervals.

As illustrated in Figure 1, experienced parental violence was the single input variable with the highest predictive value for sexual violence victimizations, *p* < 0.001, with a prevalence rate of 4.0% (CI_95%_ (3.15; 4.85)). For those women who had experienced at least one incident of “minor” parental violence as a child, experienced contact CSA gave the most subsequent information, *p* < 0.001: If women had additionally been sexually abused as a child, the five-year prevalence of sexual violence rose to 8.6% (CI_95%_ (5.24; 11.96)), as compared to 3.3% (CI_95%_ (2.48; 4.12)) if they had experienced parental violence, but not contact CSA. By far the highest prevalence rate, that is, 17.0% (CI_95%_ (6.26; 27.74)), was found for women who had experienced both parental violence as well as contact CSA and were currently divorced, separated, or widowed (Subgroup 7), *p* = 0.023. Subgroup 6 with an increased victimization risk of 6.8% (CI_95%_ (3.48; 10.12)) represents women with a history of parental violence and contact CSA who were currently not divorced, separated, or widowed. Subgroups 4 and 5 include women who had experienced parental violence, but not contact CSA, and who either lived together with others (Subgroup 4), 2.7% (CI_95%_ (1.89; 3.51)), or on their own (Subgroup 5), 6.5% (CI_95%_ (3.76; 9.24)), *p* < 0.001.

In the left part of Figure 1, the decreased prevalence rate for women without a history of parental violence, 1.1% (CI_95%_ (0.68; 1.52)), and the respective subcategories are depicted. In this case, knowing about the penalization of marital rape gave the most subsequent information, *p* = 0.002: For those women without a history of parental violence who indicated that they currently knew that marital rape is as prosecutable as rape by any other person, the victimization risk decreased even further to 0.9% (CI_95%_ (0.49; 1.31)). In comparison, women who had not experienced parental violence and were not aware of the current legal situation (Subgroup 1), had a prevalence rate of 2.9% (CI_95%_ (0.92; 4.88)). The lowest victimization risk was predicted for Subgroup 2, that is, women with no history of parental violence who knew about the penalization of marital rape and who currently did not live on their own, *p* = 0.004, 0.6% (CI_95%_ (0.23; 0.97)). Again, living alone was associated with an increased victimization risk—in this case even for women without history of parental violence who knew that marital rape is prosecutable (Subgroup 3), 2.1% (CI_95%_ (0.65; 3.55)).

Summarizing the survivors in the seven subgroups as depicted in Figure 1, results in the total number of *n* = 110 victims of sexual violence within the previous five years in this sample. The remaining predictors (age, educational degree, origin, and residential area) did not significantly contribute to the C-Tree analyses beyond the predictors outlined above.

## 4. Discussion

### 4.1. Summary and Research Implications

The present study yielded important information about the current prevalence of sexual violence against women in Germany—especially accounting for factors associated with an increased or decreased prevalence of experienced sexual violence. Summarizing briefly, more than 1 in 20 women aged 21 to 40 years indicated that they had experienced sexual violence in her life, 1 in 40 women had been victimized within five years prior to data collection. Regarding the five-year prevalence of experienced sexual violence, bivariate analyses revealed associations with prior childhood victimizations, self-reported knowledge about the penalization of marital rape, and several sociodemographic characteristics, such as household size, relationship status, and level of education. In order to identify specific risk markers while simultaneously accounting for interactions between the predictors, C-Tree analyses were applied. These analyses confirmed experienced parental violence and contact CSA before the age of 16 years as most important risk markers for sexual violence victimizations in adolescence and adulthood, characterizing by and large 76.4% of the survivors in the present sample.

#### 4.1.1. Prior Victimization

It is important to note that C-Tree analyses are capable of detecting the exact threshold of a significantly increased risk in non-dichotomous variables. That is, with respect to parental violence, only one incident of “minor” violence by father, mother, or other primary caregiver before the age of 16 years was associated with an increased five-year prevalence rate of sexual violence by a factor of four as compared to women who had experienced no single incident of parental violence as a child. The already increased victimization risk, in turn, doubled if contact CSA had additionally been experienced in childhood. Taken together, the present results obtained through an advanced statistical method confirm that childhood victimizations need to be considered as major risk factors for experiencing sexual violence in adolescence and adulthood.

As the link between childhood victimizations and sexual (re)victimization later in life has already been mentioned in previous research, the present study convincingly contributes to the growing body of research in this domain (e.g., [27,33,38]). Yet, potential explanations and underlying processes for this link remain unclear. Growing up in a dysfunctional family environment is, for example, related to adverse developmental outcomes, such as negative self-image and emotional dysregulation [40,71]. These outcomes are further (directly and indirectly) related to risky behaviors, such as substance abuse or a high-risk sexual lifestyle, which in turn can further contribute to an increased risk of sexual (re)victimization [31,32,34,40,72]. Through these subsequent links, initial physical and sexual abuse in childhood may predispose and create a high-risk (re)victimization environment (see [38]). However, these processes do not indicate a causal relationship between prior victimization and later (re)victimization due to the victims’ behaviors that could in any way make them responsible for the victimization. In sum, future research needs to further focus on the link between experienced parental violence, CSA, and (re)victimization in adolescence and adulthood [31,36,73], while accounting for other forms of child maltreatment as well as biasing factors, such as social desirability in self-reports [39,41].

Furthermore, it might be of interest to take into account protective factors within the family and other close social environment. Tillyer and colleagues [74], for instance, found that attachment to parents and to peers reduced the risk of sexual assault at school. Against this backdrop, it seems conceivable that the negative link between childhood victimization and sexual (re)victimization in adolescence and adulthood is paralleled by a protective function of affection and support by parents and other closely related persons [42].

#### 4.1.2. Sociodemographic Characteristics

The present results add to existing research [23,38,45] as those constellations were specified in which household size and relationship status are in fact related to an increased risk of sexual victimization: Currently being divorced, separated, or widowed was only associated with an increased prevalence rate if women had a history of parental violence and contact CSA. Similarly, living alone predicted an increased victimization risk for women who had experienced parental violence (but not contact CSA) and women without a history of childhood victimizations who knew about the penalization of marital rape. Naturally, in this cross-sectional design, we cannot rule out that the survivors’ current sociodemographic characteristics differed from those at the time of the victimization. Thus, we used the five-year prevalence rate instead of the lifetime prevalence as response variable in the C-Tree analyses in order to minimize this possibility. However, it is also possible that the sexual violence victimization preceded the aforementioned variables or even caused respective changes, such as separation or divorce, following the victimization.

The present study attempted to disentangle the relevance of sociodemographic variables as risk markers regarding sexual violence victimizations. Providing pivotal evidence in this regard, this might encourage future research to more specifically analyze the details of specific risk and protective factors as well as to further examine the underlying mediating and moderating processes against the backdrop of lifestyle-routine activities theory. The FRA report, for example, comprehensively depicts the situation of women in Europe regarding gender-based violence and outlines different possible explanations that may account for the observed differences in prevalence rates for sexual violence victimizations among the 28 participating EU Member States [5]. These explanations may, for instance, serve as starting points for future research additionally applying advanced statistical methods.

#### 4.1.3. Knowledge About the Penalization of Marital Rape

Self-reported knowledge about the current legal situation regarding marital rape was related to a decreased victimization risk. Knowing that marital rape is as prosecutable as rape by any other person may thus be understood as protective factor against sexual violence victimizations. Against the backdrop that most sexual violence victimizations happen in the survivors’ immediate social environment [5,8,59], knowledge about the penalization of marital rape may be linked to acknowledging an incident of sexual “contact” against the survivor’s will as sexual violence or rape. Based on their meta-analysis, Wilson and Miller [75] estimated a mean prevalence rate of unacknowledged rape of about 60%. Importantly, Littleton [76] found that unacknowledged sexual violence victims’ revictimization risk was almost twice as high in comparison to those victims who acknowledged what had happened to them as rape. Possessing a (more or less) clear concept of which behaviors constitute (prosecutable) sexual violence victimizations thus may have a protecting value—at least with respect to revictimization. Since acknowledgment of sexual violence victimization gradually develops with time [75], psychoeducation might be a suitable prevention measure as outlined below.

However, according to the present results, the protective function of knowledge about the current legal situation regarding marital rape only worked for those women who had not experienced a single incident of parental violence as a child. This raises the question of which factors (e.g., of the respective women’s families or social environment) are basically determinative for this knowledge. Therefore, future research should consider possible explanatory variables for this effect. However, the applied C-Tree analyses could already rule out participants’ level of education as potentially explaining variable in this regard.

### 4.2. Implications for Prevention

Although we discuss “risk markers” for experiencing sexual violence in the present paper, any form of victim blaming is defied [24,25], as we do not deny at all that offenders are responsible for victimizations. Obviously, women’s behaviors are not causally related to experienced sexual violence. Examining individual and situational victim characteristics that possibly increase the risk of sexual violence victimizations can help to identify specific constellations associated with an increased likelihood of such victimizations and therefore contribute to the comprehension of vulnerability patterns. However, we emphasize that prevention measures should set a main focus on the *offenders’* attitudes and behaviors, as they are responsible for the victimization and the survivors are not [77,78]. Yet, as the present research involves solely (non-)victims’ data and no data regarding perpetration or offenders’ motivation, we derive implications for prevention by primarily taking the (potential) victims’ perspective in the following.

Because the present analyses revealed *one single incident of “minor” parental violence* before the age of 16 years as the main risk factor for experiencing sexual violence in adolescence and adulthood, primary prevention should address this point, for example by reducing the pervasiveness of corporal punishment through large public campaigns and promote healthy interpersonal relationships [6,79]. The home visiting program for low-income, first-time mothers “Pro Kind”, for instance, was implemented successfully in parts of Germany and is based on the U.S. Nurse-Family Partnership program [80]. This program especially focusses on early childhood, as trained assistants accompany mothers from pregnancy until the child’s second birthday. Thus, early indicators for abusive potential as well as unbearable stress for the parents that might potentially foster abuse can be identified and intervening measures can be initiated. Furthermore, mothers’ own negative childhood experiences may be taken into account if applicable.

As outlined above, physical abuse and CSA are associated with an increased likelihood of risky behaviors—for example, the number of sexual partners [30,34] or substance abuse [29,32]—which may in turn relate to an increased (re)victimization risk. Therefore, in secondary prevention, not only current (potentially risky) behaviors should be addressed (e.g., through common risk reduction interventions), but also additional factors that can promote revictimization as well as a survivor’s prior victimization history need to be taken into account.

With respect to the present study’s results, psychoeducation may constitute a starting point for primary as well as secondary prevention measures. Since it was found that knowledge regarding the penalization of marital rape was associated with a decreased victimization risk, one means of prevention may focus on specific information campaigns in this regard. Bridges and colleagues [81], for instance, showed that a brief psychoeducative intervention in the domain of intimate partner violence significantly increased participants’ correct identification of respective scenarios. In a related vein, Laun [82] found that psychoeducation enhanced both sexual and domestic violence victims’ mental health. Psychoeducation regarding recognition as well as acknowledgement of sexual violence, for example, might thus be suitable for primary and secondary prevention of sexual violence victimization as well as for interventions in this domain [77,83]. Obviously, psychoeducation with respect to appropriate behavior, interpretation of seemingly ambiguous situations, the unmistakable meaning of “no” as well as the consequences of experienced sexual violence for the survivors among other things constitutes a prevention measure of utmost importance for (potential) offenders.

### 4.3. Limitations

Despite the size and representativeness of our sample, some limitations have to be addressed. Firstly, the present analyses included female respondents only. Because men experience sexual violence as well, although undeniably to a lesser extent than women, future research should also consider this often neglected group. Furthermore, future research might additionally take into account sexual violence victimizations by female offenders [84,85]. Secondly, we focused on non-institutionalized persons only, although the phenomenon of prison rape, for example, is an acknowledged problem and needs to be considered further [86]. The same reasoning applies to other high risk groups (e.g., persons with disabilities).

Furthermore, it is important to note that cross-sectional data cannot disentangle the temporal order between victimization and current sociodemographic variables, such as being divorced, or detect causal relationships. Although demographic indicators are typically used as proxies for particular lifestyles or routine activities, openness to alternative explanations is mandated (e.g., [23]).

Moreover, we exclusively relied on self-reports. Victims of violence may underreport their victimizations due to various reasons (e.g., shame, feelings of guilt, fear, or loyalty). Additionally, it might seem socially desirable to indicate knowledge about the current legal regulations regarding marital rape. Although we aimed at minimizing biased responding, for example through the sealed envelope technique [87], these limitations have to be kept in mind when interpreting the present results [88].

Importantly, the use of a single-item measure to capture sexual violence victimizations does not correspond to the state of the art. According to the World Health Organization [89], the gold standard in assessing violent victimizations pertains to direct, behaviorally-related questions as opposed to global questions. Additionally, using a single-item measure might have led to underestimated prevalence rates [90,91]. Although we used a single-item measure in the present study, the utilized question was comparatively broad: Not only were cases of completed as well as attempted rape included, but also enforced “similar activities”. In doing so, we aimed at capturing experienced sexual violence in adolescence and adulthood in a comparatively broad sense. As outlined above, a similar shortcoming concerns the assessment of knowledge regarding the penalization of marital rape, because the item formulation can be perceived as leading and thus fostering socially desirable responding. Thus, in addition to the explorative application of this factor into the C-tree analyses, the item’s imperfect wording needs to be taken into account when interpreting the result.

Of course, the present study’s results have to be interpreted in light of the respective cultural setting. Diehl and colleagues [92], for instance, referred to Hofstede’s cultural dimensions theory [93] and the global gender gap index [94] in order to relate their results to other cultural settings. These authors conclude that one may provisionally declare that Germany does not differ very much from other European Countries or the USA with respect to gender-related attitudes and societal development. However, especially with respect to differences in the legal context of marital rape (e.g., [95,96]), it would be interesting to conduct a respective victim survey and corresponding statistical analyses in other countries in order to see whether the presently identified risk and protective markers replicate in different cultural contexts.

## 5. Conclusions

This research revealed reliable results to depict the epidemiology of sexual violence against women in Germany as well as crucial risk markers in this regard. As both physical and sexual abuse during childhood considerably elevate the risk of subsequent sexual violence victimization, it has to be a foremost concern regarding victim prevention programs to focus on early child victimizations as outlined above to break the circle of (re)victimization which can perpetuate itself throughout an entire life.

## Figures and Tables

**Table 1:** Descriptive statistics for all variables used in the C-Tree analyses.

Caption	Missings (%) ^a^	Range	Mean ^b^
Experienced sexual violence	0.7	0–1	0.02
Age	0	21–40	30.64
Educational degree	1.8	1–3	2.14
Household size	0.6	1–5	2.80
Origin	0	1–3	1.29
Relationship status	0.6	1–3	1.59
Residential area	0	1–3	1.83
Experienced contact CSA	0.5	0–1	0.08
Experienced parental violence	0.7	0–2	0.62
Knowledge about the penalization of marital rape	0.8	0–1	0.87

^a^ Missings before imputation; ^b^ Means after imputation.

**Table 2:** Effect sizes (relative risks) in cases of presence and not-presence of the risk markers used as predictors in the C-Tree analyses.

Caption	Exposed		Not Exposed		RR	*p*
Risk Markers	SV	Non-SV	SV	Non-SV		
PV	84	2020	26	2320	3.60 [2.33–5.57]	<0.001
PV + CSA	21	247	89	4093	4.12 [2.65–6.42]	<0.001
PV + CSA + Divorced	8	39	102	4301	7.35 [3.79–14.21]	<0.001
PV + CSA + Not divorced	15	206	95	4054	3.02 [1.78–5.12]	<0.001
PV + No CSA	61	1775	49	2565	1.77 [1.22–2.57]	0.025
PV + No CSA + Living alone	20	290	90	4050	2.97 [1.85–4.75]	<0.001
PV + No CSA + Not living alone	41	1485	69	2855	1.14 [0.78–1.67]	0.505
No PV	26	2320	84	2020	0.28 [0.18–0.43]	<0.001
No PV + Legal knowledge	18	2302	92	2038	0.18 [0.11–0.30]	<0.001
No PV + Legal knowledge + Living alone	8	369	102	2971	0.64 [0.31–1.30]	0.218
No PV + Legal knowledge + Not living alone	10	1683	100	2657	0.16 [0.09–0.31]	<0.001
No PV + No legal knowledge	8	268	102	4072	1.19 [0.58–2.41]	0.637

Note: SV = Sexual violence; RR = Relative risk; PV = Experienced parental violence; CSA = Experienced contact child sexual abuse.
